# A kinome-targeted RNAi-based screen links FGF signaling to H2AX phosphorylation in response to radiation

**DOI:** 10.1007/s00018-015-1901-7

**Published:** 2015-04-18

**Authors:** Sami Benzina, Amandine Pitaval, Claudie Lemercier, Celine Lustremant, Vincent Frouin, Ning Wu, Alexandre Papine, Françoise Soussaline, Paul-Henri Romeo, Xavier Gidrol

**Affiliations:** 1 CEA, IRTSV, Biologie à Grande Echelle, 17 rue des Martyrs, 38054 Grenoble Cedex, France; 2 INSERM, U1038, Grenoble, France; 3 Université Grenoble-Alpes, Grenoble, France; 4 CEA, DSV, IRCM, 18 route du panorama, 92265 Fontenay-Aux-Roses, France; 5 IMSTAR, 60 rue Notre Dame-des-Champs, 75006 Paris, France

**Keywords:** DSB, γH2AX, FGFR4, JNK1, ATM

## Abstract

A general radioprotective effect by fibroblast growth factor (FGF) has been extensively described since the early 1990s; however, the molecular mechanisms involved remain largely unknown. Radiation-induced DNA double-strand breaks (DSBs) lead to a complex set of responses in eukaryotic cells. One of the earliest consequences is phosphorylation of histone H2AX to form nuclear foci of the phosphorylated form of H2AX (γH2AX) in the chromatin adjacent to sites of DSBs and to initiate the recruitment of DNA-repair molecules. Upon a DSB event, a rapid signaling network is activated to coordinate DNA repair with the induction of cell-cycle checkpoints. To date, three kinases (ATM, ATR, and DNA-PK) have been shown to phosphorylate histone H2AX in response to irradiation. Here, we report a kinome-targeted small interfering RNA (siRNA) screen to characterize human kinases involved in H2AX phosphorylation. By analyzing γH2AX foci at a single-nucleus level, we identified 46 kinases involved either directly or indirectly in H2AX phosphorylation in response to irradiation in human keratinocytes. Furthermore, we demonstrate that in response to irradiation, the FGFR4 signaling cascade promotes JNK1 activation and direct H2AX phosphorylation leading, in turn, to more efficient DNA repair. This can explain, at least partially, the radioprotective effect of FGF.

**Electronic supplementary material:**

The online version of this article (doi:10.1007/s00018-015-1901-7) contains supplementary material, which is available to authorized users.

## Introduction

As early as 1991, an autocrine effect of fibroblast growth factor (FGF) in the repair of radiation damage was reported [1], and soon after the same group showed that FGFs protected endothelial cells against radiation-induced programmed cell death [2, 3]. Since then, it has been confirmed by different groups that FGF inhibits radiation-induced apoptosis in HUVEC [4, 5], hair follicle [6], and rat small intestine [7] cells. Thereafter, numerous papers have reported a general radioprotective effect of FGF [8–10]. However, to date, there is no real characterization of the molecular mechanisms involved in this process.

Phosphorylation of histone H2AX is one of the earliest responses to radiation-induced DNA damage [11]. There is a linear relationship between double-strand breaks (DSBs) as directly estimated from the number of γH2AX foci and the quantity of energy deposition within cells from 1 to 100 Gy [12]. Monitoring the formation of γH2AX foci is the most sensitive method of detecting DSBs and, as a consequence, is a very interesting approach for evaluating human exposure to ionizing radiation [13]. Because most anti-cancer drugs also induce DSBs, monitoring the formation of γH2AX foci has the potential for evaluating cancer progression and therapeutic efficacy [14–17].

Members of the PI3 kinase family, including ataxia telangiectasia mutated (ATM), AT and Rad3-related protein (ATR), and DNA-dependent protein kinase (DNA-PK), are the enzymes responsible for H2AX phosphorylation at a serine located four residues from the carboxyl terminus [18–20]. However, γH2AX-positive cells are also detected in the skin and kidneys of mice lacking either ATM or DNA-PK, suggesting that these kinases are not the only ones to phosphorylate H2AX in vivo [21, 22]. Thus, H2AX is a target of JNK signaling and appears to trigger apoptotic DNA fragmentation [23]. Similarly, serum starvation induces p38-dependent H2AX phosphorylation to induce apoptosis [24]. Together, these results suggest that different signaling cascades could be involved in the phosphorylation of H2AX. Therefore, we hypothesized a possible link between the radioprotective effect of FGF and the FGF receptor signaling pathway with the phosphorylation of H2AX. To address this issue, we exhaustively characterized all human kinases involved in the phosphorylation of H2AX.

Several studies have highlighted the power of large-scale RNA interference (RNAi) screening approaches to characterize the relevance of specific genes in signaling cascades. Novel loci required for JAK/STAT signaling have been identified by genome-wide RNAi in *Drosophila* cells [25]. A global view of receptor tyrosine kinase (TRK) signaling through the extracellular signal-regulated kinase (ERK) pathway was obtained thanks to an RNAi-based genome-wide screen in *Drosophila* cells [26]. Similarly, all human kinases and phosphatases involved in apoptosis in humans have been characterized through a systematic RNAi screen [27]. Signaling networks controlling the Golgi apparatus in human cells have also been characterized through RNAi screening [28]. Signaling cascades for DNA damage-associated G1 checkpoint responses were also identified [29]. Furthermore, these RNAi-based screens may aid in the identification of low-frequency genetic changes that can contribute to oncogenesis. Indeed, some authors have estimated that individual mutations in as many as 20 % of all human kinases can play an active role in tumorigenesis [30]. Therefore, by mimicking loss-of-function mutations, kinome-targeted RNAi-based screens may help to identify low-frequency oncogenic genetic changes in the human kinome.

Here, we used siRNA microarrays [31, 32] to perform a kinome-targeted RNAi-based screen to exhaustively characterize all human kinases involved in the phosphorylation of H2AX in response to irradiation in skin cells. We monitored, at the single-cell level, the effect of siRNA-dependent specific inhibition of approximately 650 human kinases on H2AX phosphorylation in response to irradiation in human skin cells. We have identified 46 kinases that directly or indirectly participate in the formation of γH2AX foci. Strikingly, several of these kinases belong to the FGF receptor signaling pathway.

## Materials and methods

### siRNA library

The Human Kinase siRNA set contained 1292 siRNAs targeting 646 kinases and kinase-associated genes (Qiagen, Valencia, CA, USA, Cat. no.: 1027091). This siRNA library set was designed using an informatics algorithm against all known human kinases, which ensures highly efficient knockdown in cells. The library was synthesized with two siRNA duplexes for each gene. As a negative control, an siRNA without homology to any known mammalian gene was used (AllStars negative control, Qiagen, Valencia, CA, USA). As a positive control, an siRNA targeting ATM kinase was chosen (sense: 5′-CUUAUUCAUUAGUAAUUUAdTdT-3′; antisense: 5′-UAAAUUACUAAUGAAUAAGdTdT-3′).

### siRNA microarray printing

**Fig. 1:**
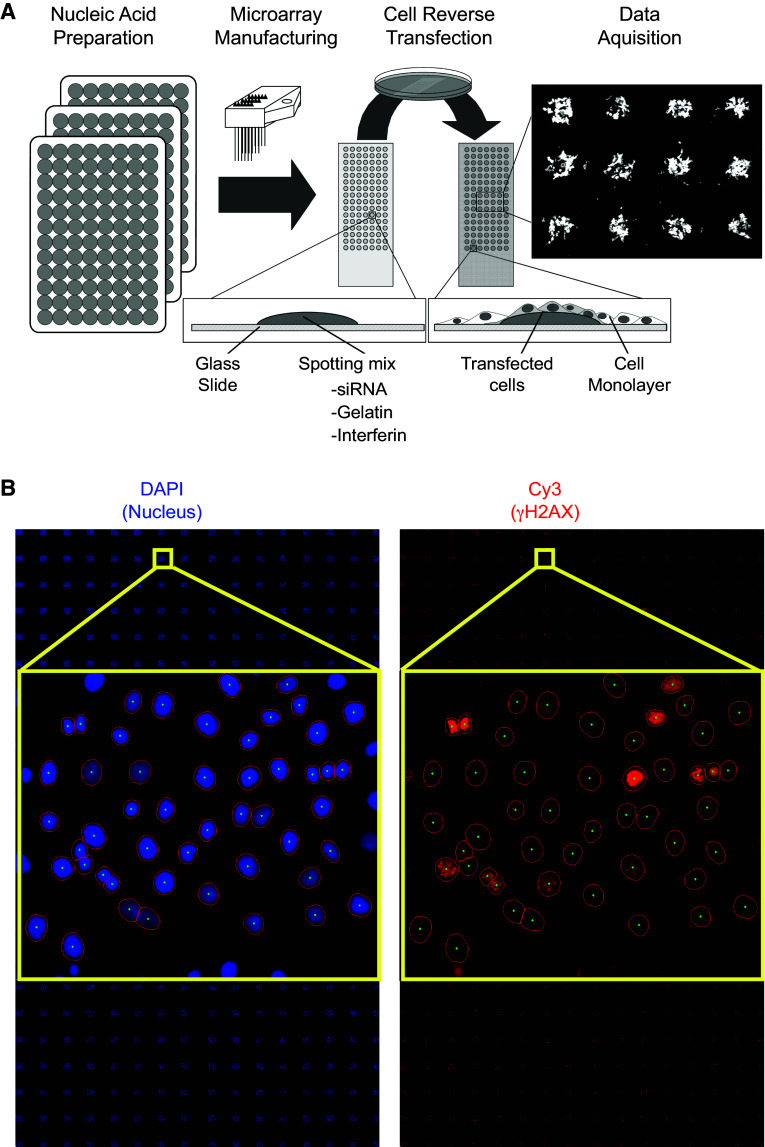
siRNA microarray. **a** HaCaT cells were grown on slides spotted with an siRNA library targeting the human kinome. Each spot on the microarray contained one specific siRNA. Forty-eight hours after transfection, the cells were irradiated at 2 Gy. Thirty minutes after irradiation, the cells were fixed and stained with DAPI and a γH2AX antibody. **b** Nuclei were stained in *blue* by DAPI and γH2AX was stained in *red* (Cy3-labeled specific antibodies). Images were automatically captured with an IMSTAR platform (Paris, France). γH2AX-specific fluorescence intensity was measured for each nucleus (DAPI) and quantified automatically

**Fig. 2:**
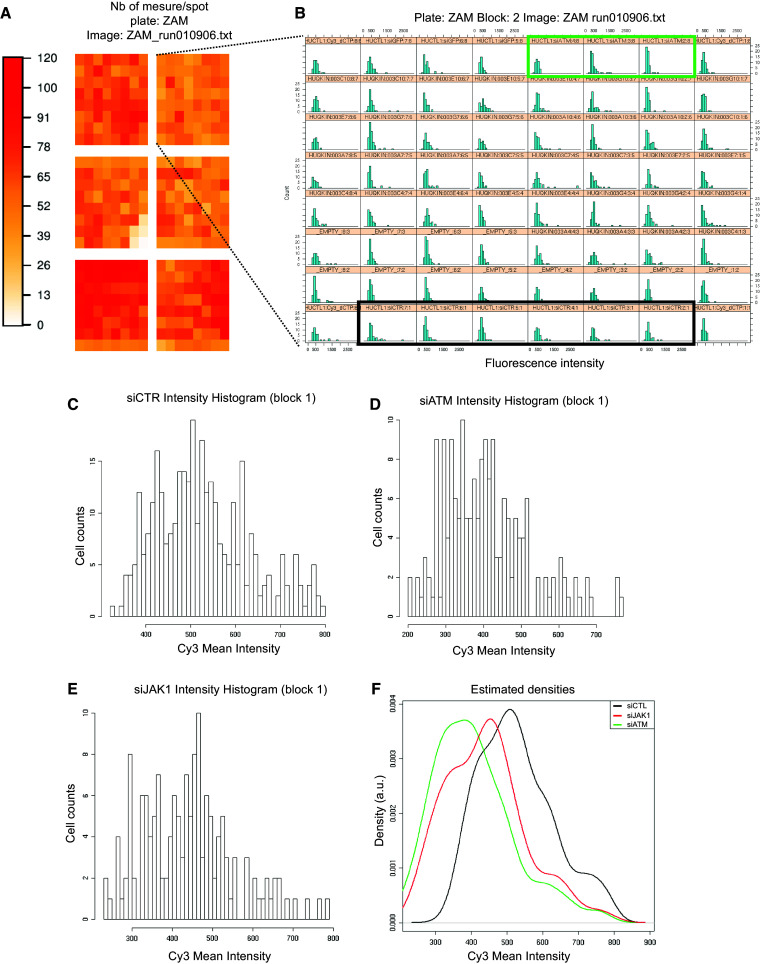
Data analysis. **a** Schematic representation of the siRNA microarray. Each spot on the microarray is schematized by a *colored square*. The *color scale on the left* reflects the number of cells on each spot. **b** Individual histograms for each of the 64 spots in block 2 of the microarray. Higher resolution areas of the histograms correspond to the negative control siRNA (**c**), ATM-targeted siRNA as the positive control (**d**) and JAK1-targeted siRNA (**e**). **f** *Density curves* generated from the histograms presented in **c**–**e**

The general procedure for cell microarray manufacturing was based on Ziauddin and Sabatini’s work [33]. As described in Fig. 1a, optimization was necessary to improve siRNA transfection into the cell line used here and to achieve good reproducibility of siRNA transfection. The siRNA set was provided in seventeen 96-well plates. The siRNA-polymer transfection solution was prepared in seventeen 96-well plates used for microarray printing (or seventeen batches of slides). In each well, 0.5 µl of siRNA duplexes (20 µM) of each target was mixed successively with 10 µl of phosphate-buffered saline and 2 µl of a 1.5 M sucrose solution. This mixture was supplemented with 2 µl of transfection reagent (INTERFERin, Polyplus-transfection, Illkirch, France). After a 10-min incubation at room temperature, 3 µl of a 1 % gelatin solution (Sigma G-1393 diluted in deionized water) and 3 µl of Matrigel™ (BD Biosciences, San Jose, CA, USA) were added in succession. The siRNA-polymer solution was arrayed in triplicate onto Superfrost Plus slides (Menzel-Gläser, Braunschweig, Germany) using a Microgrid II Biorobotics (Cambridge, UK) microarrayer at room temperature with six Biorobotics pins. The spots were 500 µm in diameter, and the dot spacing was 1100 µm. The siRNA microarray was composed of six blocks of 8 × 8 spots. Each block contained negative and positive controls (Figs. 1b, 2a). With this condition, 384 features per microarray were printed. Each siRNA-targeted kinase was arrayed in triplicate. However, an occasional spot may be missing on a microarray due to dysfunction of a printing pin. We manufactured a total of five siRNA microarrays for the study, which were stored at room temperature in a dry atmosphere until they were further processed.

### Cell culture and reverse transfection for the arrays

The human keratinocyte cell line HaCaT was generously provided by N. E. Fusenig (German Cancer Research Center, Heidelberg, Germany). HaCaT cells were grown at 37 °C in a 5 % CO_2_ humidified atmosphere in calcium chloride-free Dulbecco’s modified Eagle’s medium containing 4.5 g/l glucose supplemented with 10 % (v/v) fetal calf serum, 100,000 U/l penicillin, 50 mg/l streptomycin, and 200 mM l-glutamine. The cells were seeded at 50,000 cells/cm^2^ onto RNAi microarrays in 100-mm Petri dishes. The cells were reverse-transfected by growing them on the siRNA spot without further manipulation. Twenty-four hours after seeding, the culture medium was removed and replaced by 10 ml of preheated medium. Cells reached 90 % confluence 48 h after transfection. The entire experiment was repeated on five microarrays.

### Exposure of cell cultures to ionizing radiation

Two days after reverse or standard transfection, the HaCaT cells were irradiated with 2 Gy from a ^137^Cs source (IBL 637, CisBio International, Saclay, France) at a dose rate of 1.724 Gy/min or the Anémone/Bio irradiator (60Co, 2 Gy/min) in the ARC-Nucléart facility at CEA-Grenoble. After irradiation, the cultures were returned to the incubator. Thirty minutes later, the reverse-transfected cells were fixed in ice-cold methanol for 1 min and then washed three times with phosphate-buffered saline and stored at 4 °C. Standard transfected cells were lysed after 30 min of incubation using RIPA buffer (50 mM Tris–HCl, pH 7.4, 1 % Nonidet P-40, 0.25 % sodium deoxycholate, 150 mM NaCl, 1 mM EDTA, 1 mM Na_3_VO_4_, and 1 mM NaF) supplemented with 1× protease inhibitors (complete EDTA-free, Roche, Indianapolis, IN) to extract total proteins.

### siRNA microarray image acquisition and analysis2

Forty-eight hours after cell transfection, siRNA microarrays were irradiated at 2 Gy, and cells were fixed 30 min later. γH2AX foci formation was monitored by Cy3-labeled immunofluorescence, and nuclei were stained with DAPI. To measure γH2AX fluorescence in the nuclei of transfected cells, images were acquired with a Pathfinder OSA instrument developed by IMSTAR (Paris, France) and an automated optical scanning system equipped with a high resolution (1300 × 1000 pixels) CCD camera with a dynamic range of 12 bits or 4096 levels of intensity per pixel, coupled to a Nikon fluorescence microscope (Nikon Corporation, Tokyo, Japan). The significance of the read-out and metrics used was first verified in human HaCaT cells irradiated at 2 Gy and compared to non-irradiated cells (Fig. S1A). Because of the nucleus-to-nucleus variability of γH2AX-specific fluorescence, we compare histograms of γH2AX-specific fluorescence at the single-nucleus level from the entire cell population in triplicates rather than the averaged γH2AX signal (Fig S1B). The Pathfinder OSA instrument was programmed to visit each transfected cell spot on the siRNA microarray, perform an autofocus with DAPI, and automatically and uniformly acquire Cy3 images at a magnification of 40× with a final resolution of 0.34 µm/pixel. Two-channel images (DAPI and Cy3) were stored as frames of 16-bit Tiff images in a built-in image database (Fig. 1b). Each image contained approximately 60 HaCaT cells. Because each siRNA targeting a given kinase was spotted in triplicate on the microarrays, approximately 180 cells were analyzed per siRNA (Fig. 2a), and two different siRNAs were used per kinase. The nuclei of all of the cells in a spot were detected and quantified in the DAPI channel, and the nuclear area was automatically segmented and contoured. Using the Cy3 channel, the total fluorescence intensity of γH2AX staining in each nucleus was calculated. To compare the putative effect of a given siRNA on H2AX phosphorylation, we built fluorescence mean intensity histograms (Fig. 2b). Histograms of γH2AX-specific fluorescence at the single-nucleus level were obtained from the entire cell population in triplicate (Fig. 2c–e). Density curves were then automatically generated from the histograms (Fig. 2f). The density curve obtained for each siRNA was compared to the negative control density curve of cells transfected with an siRNA having no known target in the human genome and to the positive control density curve of an siRNA specifically targeting ATM, a kinase known to phosphorylate H2AX (Fig. 2f). Only when both controls behaved as expected in one block on the microarray were the siRNAs spotted in that block further analyzed. The density curve shift for each siRNA was then compared to the negative control density curve, and the significance of the shift was then further assessed by Mann–Whitney *U* test, which is a statistical test of the null hypothesis that two populations are the same against an alternative hypothesis. The siRNAs that showed a significant shift (*p* value <1 %) of their density curve were considered potential hits. As demonstrated in Table 1, to further increase the statistical robustness of the screening procedure, we performed five independent biological replicates (5 siRNA microarrays). Therefore, all together we had up to 15 replicates for each siRNA (sometimes fewer when spots were missing due to inefficient printing on the siRNA microarrays or not enough cells were on a spot). A kinase was considered a hit when at least one of the two siRNAs targeting that kinase passed the statistical test at least 3 times (Table 1).

**Table 1:**
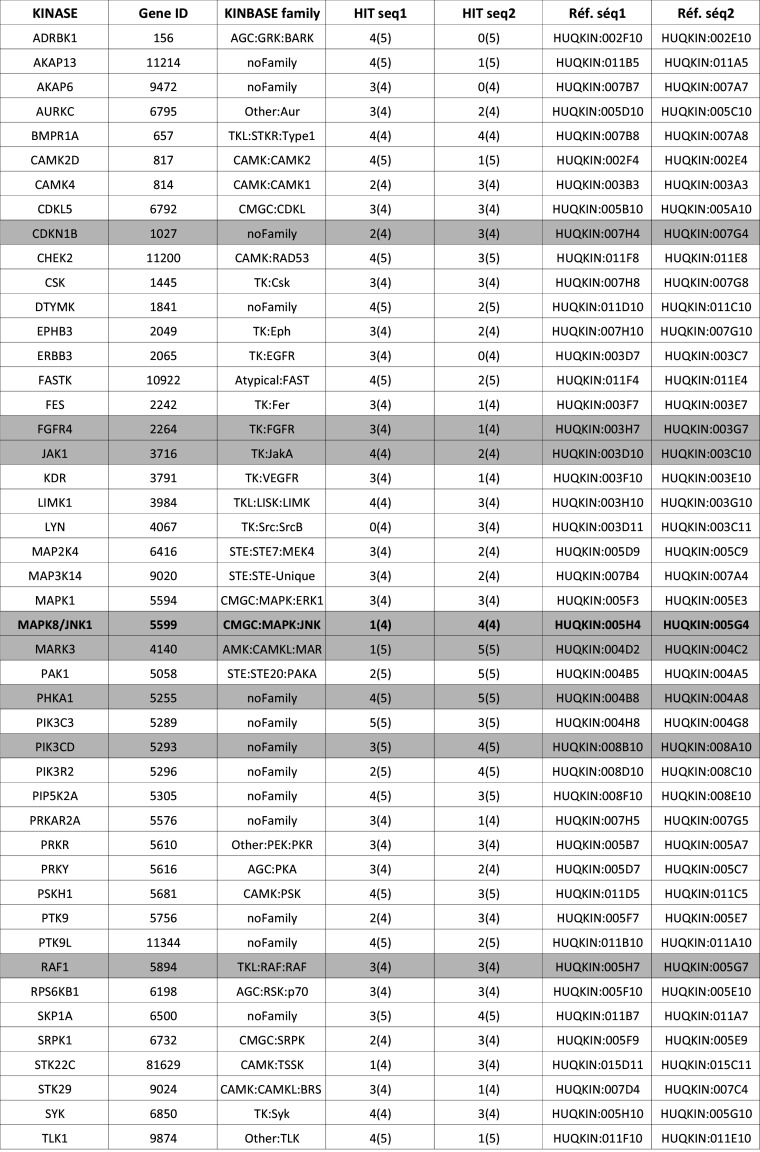
List of positive kinases identified by the genome-wide kinome-targeted screen

Ref. seq 1 and Ref. seq 2 correspond to Qiagen references (Qiagen, Valencia, CA, USA, Cat. No. 1027091) of the two siRNAs targeting each specific kinase. HIT seq 1 and HIT seq 2 correspond to the number of independent biological replicates performed with each siRNA (number between brackets). Five siRNA microarrays were used; however, an occasional spot may have been missed from the printing process or eliminated when there were too few cells on the spot. Thus, the number of repeats (between brackets) could be <5. The other number indicated how many times the siRNA being analyzed successfully passed the statistical test at a *p* value of 1 % (see “Materials and methods”). The table lists the 46 kinases for which at least one of the two siRNAs passed the test at least 3 times. Gray lines in the table correspond to kinases that were randomly chosen to be further validated in the study. Kinases already known to phosphorylate H2AX are indicated in bold. An siRNA targeting ATM was used as a positive control, and siRNA AllStar was used as a negative control

### Primary keratinocytes

Human keratinocytes were isolated from neonatal foreskin obtained from routine circumcisions. The biopsies were preserved in DMEM-PS (medium containing penicillin–streptomycin) and kept at 4 °C. Afterward, keratinocyte cell suspensions were prepared using thermolysin in combination with trypsin–EDTA. Biopsies were kept in PBS containing 0.5 mg/ml thermolysin at 4 °C for 16–20 h. Subsequently, the dermis and epidermis were separated with a pair of forceps, and the epidermis was incubated for 60 min at room temperature in PBS containing 0.125 % trypsin and 0.05 % EDTA under magnetic stirring. Then, 10 % FCS was added, and the cells were resuspended and filtered with a 70-ml cell strainer (BD Biosciences, San Jose, CA, USA) to remove the remaining aggregates prior to counting.

### Standard transfection

The HaCaT cells (3 × 10^5^) were seeded in 5 ml of the medium described above in 25 cm^2^ plastic flasks. Twenty-four hours later, 6 µl of the 20 µM siRNA duplex and 20 µl of INTERFERin (Polyplus, Illkirch, France) were diluted in 500 µl of DMEM without fetal calf serum. Ten minutes later, the mix was added to the cells with 4 ml of fresh medium for transcription. The flasks were placed in an incubator at 37 °C and 5 % CO_2_ for 48 h. HaCaT cells were also co-transfected with either pCMV6-XL5/FGFR4 plasmid or pCMV6-XL5/Mock plasmid (Origene, MD, USA) along with an siRNA control (siCTL) or an siRNA targeting ATM (siATM). Transfection was carried out using jetPRIME^®^ transfection reagent (Polyplus transfection, Illkirch, France) according to the manufacturer’s instructions. Briefly, cells were seeded (3 × 10^5^ cells) into 25 cm^2^ flasks. Four micrograms of DNA and 50 pmol of siRNA duplex were diluted in 200 μl of jetPRIME^®^ buffer, and then 8 μl of jetPRIME^®^ reagent was added to the mixture. The transfection mixture was added to the cells after 15 min incubation at room temperature, and the medium was changed after 24 h. Finally, cells were assayed at 48 h post-transfection for the appropriate activity. The same protocol was used to transfect human ATM-deficient fibroblasts with either pCMV6-XL5/FGFR4 or pCMV6-XL5/Mock plasmids (4 μg of DNA). The culture medium was changed after 4 h of transfection.

### Primary antibodies and chemical inhibitors

The following antibodies were used in this study: anti-histone H2AX (07-627; dilution 1:2000) and anti-pSer139-H2AX (05-636; dilution 1:2000) (Upstate, Billerica, MA, USA); anti-JAK1 (3332, dilution 1:1000) and anti-JNK1 (3708; dilution 1:1000) (Cell Signaling Technology, Danvers, MA, USA); anti-FGFR4 (sc-124; dilution 1:200), anti-pThr183/Tyr185-JNK (sc-6254; dilution 1:200), and anti-ATM (sc-135663, dilution 1:200) (Santa Cruz Biotechnology, Santa Cruz, CA, USA); anti-MARK3 (1952-1; dilution 1:2000) and anti-RAF1 (1560-1; dilution 1:500) (Epitomics, CA, USA) and anti-β-actin (A3854; dilution 1:5000) (Sigma-Aldrich); anti-CDKN1B (AHZ0452, Invitrogen; dilution 1:200); PHKA1 (H00005255-A01, Abnova, Taiwan; dilution 1:500); and anti-PIK3CD (AP8020a, Abgent, CA, USA; dilution 1:500). To inhibit the JNK1 and ERK pathways, specific inhibitors from Calbiochem (Billerica, MA, USA) were used (JNK inhibitor 420119, JNK1 inhibitor negative control 420123, ERK inhibitor 328007 and ERK inhibitor negative control 328008). Stock solutions were prepared at 20 mM in DMSO, and the cells were treated with a final concentration of 25 µM of inhibitor for 2 h before irradiation. PD173074 (FGFR1 and FGFR3 inhibitor) and SU5402 (FGFR inhibitor) were purchased from Tocris and Calbiochem, respectively, and both inhibitors were used at a final concentration of 10 µM.

### Immunofluorescence

The cells were incubated with a mouse monoclonal antibody against γH2AX, diluted 1:2000 in phosphate-buffered saline supplemented with 10 % goat serum for 1 h at room temperature in a humidified chamber. Excess antibody was removed by washing the slides with phosphate-buffered saline three times. Incubation with Cy3-conjugated goat anti-mouse IgG (Jackson Immunoresearch Laboratories) was performed at 1:800 in the dark at room temperature for 30 min. After several washes, the slides were mounted in Vectashield mounting medium containing DAPI (Vector Laboratories, Burlingame, CA, USA).

### Colony-forming efficiency and colony characterization

For the analysis of colony-forming efficiency (CFE), HaCaT cells were transfected for 48 h, plated after irradiation at 2, 4, and 6 Gy at 10 cells/cm^2^ and grown for 14 days. HaCaT cells were stained and fixed with 1 % crystal violet in methanol, and the number of colonies containing more than 50 cells was counted by microscopy.

### JNK interaction

Interactions between JNK1 and H2AX were observed using a SAPK/JNK assay kit (9810, Cell Signaling Technology, Danvers, MA, USA). Briefly, HaCaT cells (control or 2 Gy irradiation) were harvested 30 min, 1, 2, or 4 h after irradiation, lysed in 1 ml of 20 mM Tris–HCl (pH 7.4, 150 mM NaCl, 1 mM EDTA, 1 mM EGTA, 1 % Triton X-100, 2.5 mM sodium pyrophosphate, 1 mM β-glycerophosphate, 1 mM Na_3_VO_4_, 1 µg/ml leupeptin, and 1 mM PMSF), and sonicated. The lysates were cleared by centrifugation (4 °C, 13,000 rpm, 10 min) and then incubated with GST-c Jun (1-89) beads overnight at 4 °C on a rocker. The next day, the beads were washed twice in lysis buffer, twice in kinase buffer (25 mM Tris–HCl pH 7.5, 5 mM β-glycerophosphate, 2 mM DTT, 0.1 mM Na_3_VO_4_, and 10 mM MgCl_2_), and resuspended in kinase buffer containing 200 µM ATP. After a 30 min incubation at 30 °C, the kinase reaction was stopped with Laemmli sample buffer and analyzed by Western blotting with specific antibodies.

### Lysing protocol and immunoblotting

Total cell extracts were prepared by lysing the cells on ice for 20 min in radioimmunoprecipitation assay (RIPA) buffer (50 mM Tris–HCl, pH 7.4, 1 % Nonidet P-40, 0.25 % sodium deoxycholate, 150 mM NaCl, 1 mM EDTA, 1 mM Na_3_VO_4_, and 1 mM NaF) supplemented with 1× protease inhibitors (complete EDTA-free, Roche, Indianapolis, IN). The lysates were subjected to four pulses of sonication to shear the DNA and centrifuged at 14,000 rpm for 10 min. The supernatants containing total proteins were then analyzed by Western blotting as described below. The samples were boiled for 5 min in 0.2 % v/v of loading buffer (Lane Marker Reducing Sample Buffer, 39000, Pierce), and equal amounts of total protein were analyzed on a 4–12 % gradient gel (NuPAGE 4–12 % Bis–Tris gel; Invitrogen). After electrophoresis, the proteins were transferred onto a nitrocellulose membrane and blocked for 1 h at room temperature in Tris-buffered saline with Tween 20 (TBST) containing 5 % nonfat dry milk. The blots were then incubated with primary antibody. After several washes in TBST, the membranes were incubated in TBST containing 5 % nonfat dry milk and a 1:5000 dilution of horseradish peroxidase-linked goat anti-rabbit or anti-mouse antibody (Upstate, Billerica, MA, USA) for 1 h at room temperature. Specific bands were visualized by chemiluminescence using an ECL Plus kit (Amersham, GE Healthcare, Buckinghamshire, UK).

## Results

### Kinome-targeted loss-of-function screen

We used siRNA microarrays to enable massively parallel reverse transfections of siRNAs into human skin cells because they are the first cells to be exposed to radiation (Fig. 1a). Using this technology, we performed a kinome-targeted siRNA-mediated loss-of-function screen to identify all human kinases involved in the phosphorylation of histone H2AX and/or the formation of γH2AX foci upon irradiation in the HaCaT cell line. We transfected 1292 siRNAs targeting 646 human kinases (two siRNAs/kinase) with an average of 70 % transfection efficacy. Forty-eight hours after transfection, the arrays were irradiated at 2 Gy, and cells were fixed 30 min later. The cells were labeled with two different fluorochromes, and the images were captured and processed with a dedicated motorized microscope. DAPI-stained nuclei and γH2AX-specific fluorescence were automatically quantified at the single-nucleus level using specifically designed algorithms (Fig. 1b, Fig. S1). This analysis of the signal at the single-nucleus level was essential for determining cell-to-cell variability in the γH2AX-specific signal within and between micropatterns on the siRNA microarrays, as reflected in Fig. S1. Histograms of γH2AX-specific fluorescence were averaged for each replicate, and the corresponding density curves were generated automatically (Fig. 2). The density curve obtained for each siRNA was compared to the control density curve obtained by cells transfected with an siRNA having no known target in the human genome (Fig. 2f). A specific siRNA targeting ATM, which is a kinase known to phosphorylate H2AX, was used as a positive control and exhibited a significant shift of the histogram toward a lower fluorescence intensity (Fig. 2b–e). For each siRNA, the shift was compared to positive and negative controls, and its significance was assessed by the Mann–Whitney test (Fig. 2f). At a *p* value of 1 %, forty-six kinases were found to have a direct or indirect effect on H2AX phosphorylation (Table 1). Among those kinases, most known kinase families were represented, but the tyrosine kinase family was the most prevalent (Table 2).

**Table 2:** Kinase families

	Kinase number	TK Family	TKL Family	AGC Family	CAMK Family	STE Family	CMGC family	CK1 Family	Atypical	Other	No Family
Kinases targeted by the Qiagen siRNA collection	646	82	38	58	59	39	59	12	23	55	221
Hits alpha 1 %	46	9	3	3	7	3	4	0	1	3	13
Hits alpha 5 %	66	12	3	4	9	6	6	2	1	4	19

Kinase families are the following: *TK* tyrosine kinase, *TKL* tyrosine kinase like, *AGC* protein kinase C family, *CAMK* calcium/calmodulin-dependent protein kinases, *STE* mitogen-activated protein kinase [MAPK] family, and *CMGC* cyclin-dependent protein kinases

### Validation of the putative kinases

**Fig. 3:**
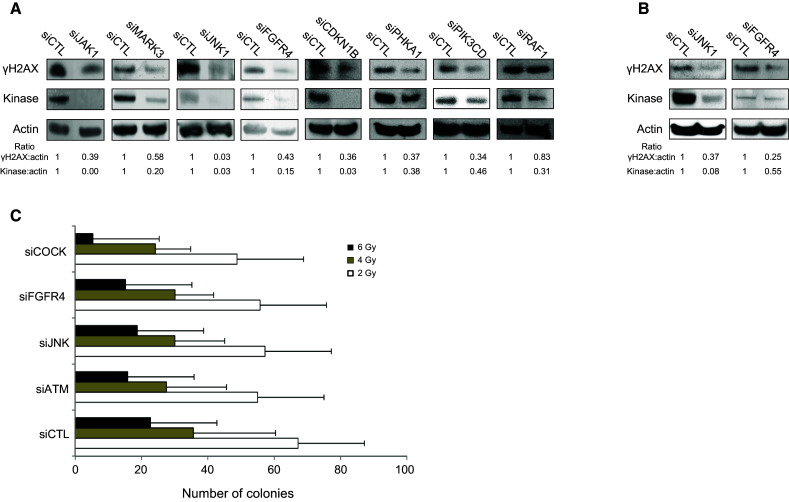
Validation of the screening results by Western blotting. **a** Eight kinases selected in the screen were randomly chosen for further validation. Seven of them exhibited reduced expression at the protein level upon siRNA-mediated knockdown and reduced phosphorylation of H2AX in HaCaT cells after irradiation at 2 Gy. **b** Knockdown of JNK1 and FGFR4 reduced the phosphorylation of H2AX in primary keratinocytes after irradiation at 2 Gy. **c** Clonogenic assay on HaCaT cells irradiated at 2, 4, and 6 Gy and previously transfected with siCTL, siATM, siJNK1, siFGFR4 or a combination of the last three siRNAs (siCOCK for cocktail). *Error bars* represent standard deviation

Eight kinases (in gray in Table 1) were randomly selected from the target list for further validation by Western blotting to confirm knockdown of the targeted kinase as well as inhibition of H2AX phosphorylation. Forward transfection of siRNA into HaCaT cells was associated with siRNA-dependent silencing of all eight kinases. RAF1-targeted siRNA was the only transfected siRNA not to efficiently reduce the level of γH2AX; therefore, one can estimate that 7/8 (88 %) of the kinases tested were experimentally validated (Fig. 3), suggesting that among the 46 targets selected in the screen, nearly 40 of the kinases are likely to be involved, either directly or indirectly, in the phosphorylation of H2AX and/or in the formation of γH2AX foci. None of the kinases but JNK1 have been reported before as having a role in the phosphorylation of histone H2AX. These results suggest that the phosphorylation of histone H2AX in response to DSBs involves many molecules including several kinases, such as FGFR4 and JAK1, which are either membrane bound or associated. The participation of JNK1 and FGFR4 in the irradiation-dependent phosphorylation of H2AX was also confirmed in human primary keratinocytes obtained from healthy donors (Fig. 3b). Finally, to confirm the participation of numerous kinases in H2AX phosphorylation, we analyzed the synergistic effect of kinase knockdown on the cytotoxic response to irradiation, evaluated by colony-forming efficiency. After irradiation at 6 Gy, the extinction of three kinases, ATM, FGFR4, and JNK simultaneously, was more toxic than the ablation of any kinase individually (Fig. 3c).

### FGF signaling participates in H2AX phosphorylation

Using the knowledge-based software Ingenuity, we identified the most prominent signaling pathways among our list of kinase “hits.” Interestingly, although we screened human HaCaT keratinocytes cells, B cell receptor (BCR) signaling was the first pathway extracted from the IPA database (*p* value = 10^−17^) (Fig. S2). As H2AX is required for efficient immunoglobulin class switching in lymphocyte B [34, 35], this result was not unexpected and indicated that kinases belonging to the BCR signaling pathways in B cells also participate in H2AX phosphorylation in cells lacking B cell receptors, such as human keratinocytes.

**Fig. 4:**
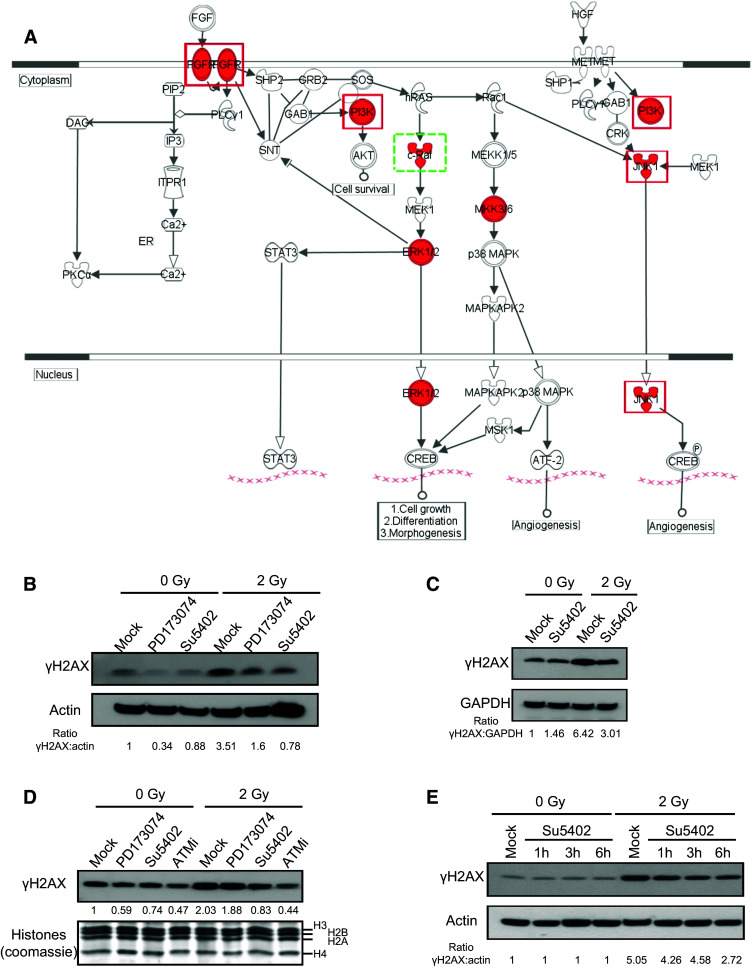
Role of FGF signaling in H2AX phosphorylation. **a** Schematic representation of FGF signaling. Kinases selected in the screen are in *red*. *Red-framed* kinases are validated by Western blotting; the *green-framed* kinase is the only protein not validated by Western blotting. **b** Inhibition of FGF signaling by specific inhibitors PD173074 and SU5402. Cells were treated for 4 h with 10 µM PD173074, SU5402, or DMSO (mock) with and without irradiation at 2 Gy for 30 min. **c** Similar to **b** using GAPDH as the loading control. **d** Similar to **b** but with histones used as the loading control; ATM inhibitor was used at 10 µM as in the control. **e** Time-course inhibition of FGF signaling by the specific inhibitor SU5402. Cells were treated for 1, 3, or 6 h with 10 µM SU5402 or DMSO (mock) with and without irradiation at 2 Gy for 30 min

Several other pathways were also identified by the IPA software, including RAC signaling (*p* value = 10^−13^) (Fig. S3) and FGF signaling (*p* value = 10^−11^) (Fig. 4a). Within the FGF signaling cascade, four kinases (FGFR4, PI3K, ERK, and JNK1) were selected in the screening process, and three were further validated. To confirm the role of FGFR4 in H2AX phosphorylation in response to DSB and to eliminate potential off-target effects, we inhibited FGF signaling with two different chemical inhibitors, PD173074 and Su45402. As shown in Fig. 4b–e, both inhibitors significantly decreased the level of phosphorylation of H2AX in response to irradiation. We also confirmed the effect of both inhibitors using alternative controls, including GAPDH (Fig. 4c) and histones (Fig. 4d). The ATM inhibitor was also used as a control and was the most efficient for decreasing H2AX phosphorylation, though it was not completely abolished (Fig. 4d). As Su5402 was the most efficient, we confirmed its ability to inhibit H2AX phosphorylation at 1, 3, and 6 h after irradiation and also observed that it does not affect the basal level of H2AX phosphorylation in the absence of irradiation (Fig. 4e). Interestingly, the fact that most inhibitors, either siRNA or chemical inhibitors (Figs. 3, 4b–e), were unable to completely abolish the γH2AX signal indirectly confirms that many kinases participate in H2AX phosphorylation in response to irradiation. Because of its membrane integration, the effect of the FGF receptor on H2AX phosphorylation was most likely indirect but could ultimately lead to increased phosphorylation of H2AX after irradiation.

### FGFR4-related phosphorylation of H2AX is independent of ATM

**Fig. 5:**
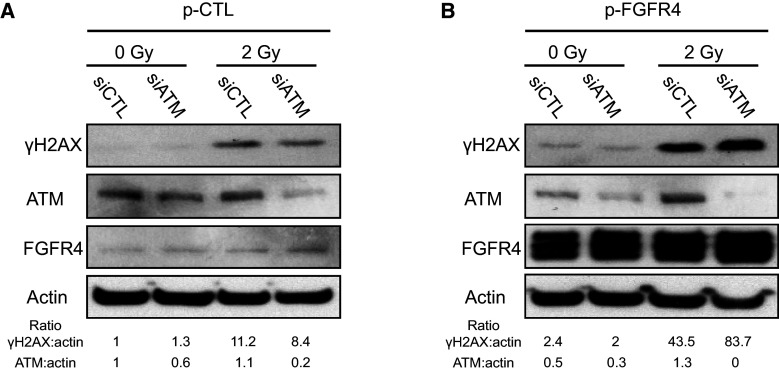
The role of FGFR4 in H2AX phosphorylation is independent of ATM. **a** HaCaT cells were transfected with pCMV6-XL5/Mock (control vector, p-CTL) or **b** pCMV6-XL5/FGFR4 (FGFR4 expression vector, p-FGFR4), along with control siRNA or ATM-targeted siRNA. The cells were then irradiated at 2 Gy. The protein extracts were analyzed by Western blotting using anti-γH2AX, anti-ATM, or anti-FGFR4 antibodies. Actin expression was used as a control. Two independent biological replicates were performed for this experiment

To evaluate the role of ATM in H2AX phosphorylation in response to FGF signaling, we overexpressed FGFR4 while knocking down ATM in HaCaT cells and monitored the phosphorylation of H2AX and the formation of γH2AX foci (Fig. 5). We observed accumulation of γH2AX in FGFR4-overexpressing cells in response to irradiation. Interestingly, FGFR4 overexpression led to an increase in γH2AX even in the absence of irradiation (Fig. 5). Phosphorylation of H2AX was reduced after ATM silencing in control-plasmid transfected cells but not in FGFR4-overexpressing cells. This result demonstrated that FGFR4 participates in the phosphorylation of H2AX via kinase(s) other than ATM. Inhibition of ATM by siRNA (Fig. 5) or specific chemicals (Fig. S4) did not completely abolish the phosphorylation of H2AX (Fig. 5b), and two peaks were clearly observed in the density curves (Fig. S4). Again, these results confirmed that ATM is not the only kinase able to phosphorylate H2AX and that downstream of FGF signaling, other kinases phosphorylate H2AX in the keratinocyte nucleus.

### JNK1 phosphorylates H2AX downstream of FGF signaling

**Fig. 6:**
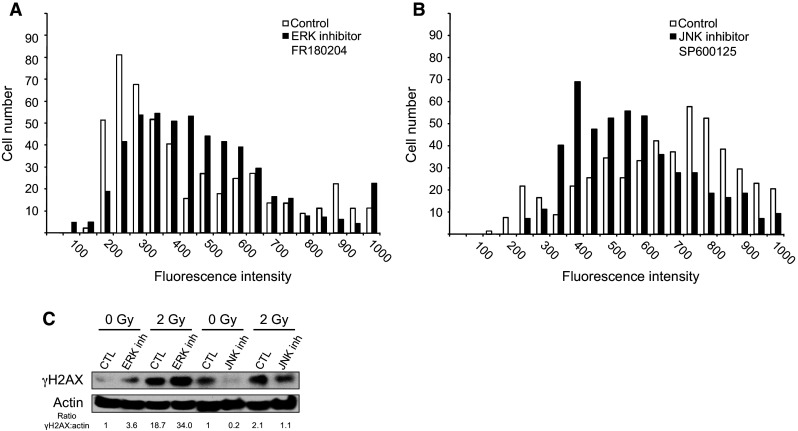
Impact of the inhibition of either ERK or JNK on H2AX phosphorylation. **a** HaCaT cells were irradiated at 2 Gy with or without an ERK-specific inhibitor and then co-stained with DAPI and anti-γH2AX fluorescent antibodies. The γH2AX-specific signal was quantified at the single-cell level. **b** Similar to A with the JNK-specific inhibitor. **c** γH2AX content was also analyzed by Western blotting with or without inhibitors and with or without irradiation. Two independent biological replicates were performed for **c**

Two candidate kinases, ERK1/2 and JNK1, which are shuttled to the nucleus upon activation of FGFR4, could potentially catalyze the phosphorylation of H2AX. To confirm their involvement in H2AX phosphorylation and to eliminate potential off-target effects, we used the chemical inhibitors FR180204 and SP600125 to specifically inhibit ERK1/2 and JNK1, respectively. Analysis of H2AX phosphorylation after irradiation in the presence of each inhibitor showed that JNK1 inhibition significantly reduced H2AX phosphorylation (Fig. 6b), whereas the ERK inhibitor had no effect (Fig. 6a). These results were confirmed by Western blotting (Fig. 6c) and demonstrated that JNK1 was a potential candidate for H2AX phosphorylation downstream of FGF signaling.

**Fig. 7:**
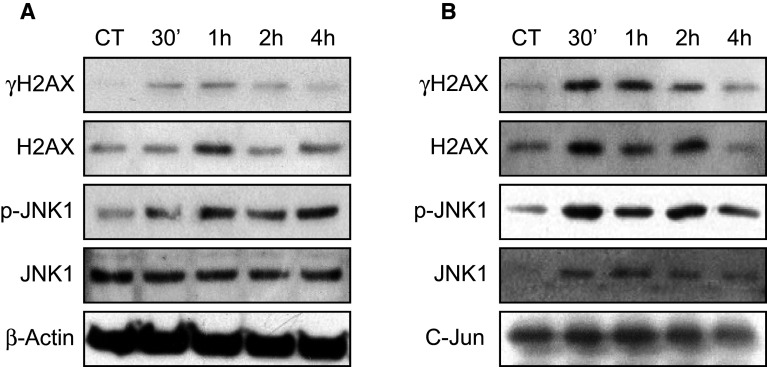
H2AX and JNK1 interaction assay. **a** The kinetics of H2AX and JNK1 phosphorylation in HaCaT cell 30 min, 1, 2, or 4 h after irradiation was directly monitored by Western blotting for γH2AX, H2AX, JNK1, and pJNK1, with β-actin used as a charge control. **b** The same protein extracts were incubated with GST-C-Jun beads to immunoprecipitate JNK1 using a SAPK/JNK Kinase Assay Kit and to monitor co-immunoprecipitated proteins. Immunoprecipitated proteins were separated by SDS-PAGE 30 min, 1, 2, or 4 h after irradiation. The presence of γH2AX, H2AX, JNK1, and pJNK1 was evaluated by Western blotting, and C-Jun was used as a charge control. H2AX and γH2AX were co-immunoprecipitated with JNK as soon as 30 min and up to 2 h after irradiation. Two independent biological replicates were performed

Finally, we analyzed the time-course phosphorylation of H2AX and JNK1 in response to DNA damage. As demonstrated in Fig. 7a, Western blots of proteins extracted at different times after irradiation show that JNK1 kinase was phosphorylated as soon as 30 min after irradiation, concomitantly with H2AX. To further explore whether JNK1 was directly involved in H2AX phosphorylation, we immunoprecipitated JNK1 using GST-c-Jun agarose beads and analyzed the co-immunoprecipitated proteins (Fig. 7b). We confirmed that JNK1 was phosphorylated as soon as 30 min after irradiation and showed that H2AX and γH2AX were co-immunoprecipitated with JNK1 and that their levels increased when JNK1 phosphorylation increased (Fig. 7b). These results suggest that in response to ionizing radiation, JNK1 could directly interact with and phosphorylate H2AX in the cell downstream of FGF signaling.

## Discussion

Phosphorylation of H2AX at DSBs allows for its participation in the repair of DSBs by cellular machinery. To date, three different kinases, ATM, ATR, and DNA-PK, have been reported to be the catalysts of H2AX phosphorylation in response to genotoxic threats [19, 20]. The kinome-targeted RNAi-based functional screen we performed demonstrates that approximately 40 kinases participate, directly or indirectly, in H2AX phosphorylation in response to irradiation. Several reasons may explain why our characterization results in such a large number of kinases. Classical approaches are unlikely to permit a comprehensive characterization of signaling networks, as they rely mostly on the analysis of specific kinases. The analysis of DNA-repair pathways relies mainly on well-characterized canonical components (e.g., ATM, ATR, and DNA-PK). Performing exhaustive and unbiased screening using non-canonical molecules, as in this study, measures signaling outputs at different points within the network and enables the discovery of new molecular targets. Furthermore, because H2AX phosphorylation was monitored at the single-cell level, we were able to analyze the distribution of the signal rather than its average, which facilitates the characterization of subtle effects of kinases.

ATM is a serine/threonine protein kinase that is recruited and activated by DNA double-strand breaks. It is key molecule in DNA repair through the phosphorylation of several targets, including p53, NBS1, and H2AX [36–38]. ATM is also the main kinase responsible for the phosphorylation of H2AX in response to ionizing radiation [39], and in our screen, no siRNA was more efficient than the ATM-targeting siRNA at decreasing H2AX phosphorylation. The ATM gene ranked third in terms of mutation frequency in a recent analysis of 518 protein kinases in 210 human cancers [30]; therefore, other kinases may compensate for ATM loss of function. However, several kinome-targeted functional screens in various organisms have shown the implication of tens of kinases in a given phenotype. Ninety kinases are involved in JAK/STAT signaling [25], and 331 RTK/ERK pathway regulators were found in Drosophila cells [26]. Seventy-three kinases were identified as survival kinases in HeLa cells [27]. Fifty-nine kinases were positive regulators of neurite outgrowth in human cells, and 66 inhibited neurite outgrowth [40]. Even more striking, the depletion of 159 signaling genes, nearly 20 % of the genes assayed, induced strong and varied perturbations in Golgi morphology [28]. Our results and those of others support the emerging view that there are not a specific number of regulators in a signaling network. The definition of the number of regulators is based instead on arbitrary thresholds [26, 41, 42]. Furthermore, our results and those of others argue against the idea that signaling pathways are limited to a few canonical components and show that large regulatory networks have a graded effect on signaling output [43], even though some key kinases can still have a major effect relative to others.

Paulsen et al. [44] conducted a genome-wide screen to identify all human genes that upon siRNA-mediated inhibition resulted in increased phosphorylation of H2AX. They identified hundreds of genes whose knockdown resulted in more γH2AX and revealed that the preservation of genome stability is mediated by a large network of genes. Our objective was complementary to this previous study in that we looked for kinases whose knockdown resulted in less γH2AX. Surprisingly, our screen revealed that membrane-associated kinases such as JAK1, or even membrane-bound kinases such as FGFR4, were involved in H2AX phosphorylation. The implication of membrane-associated proteins in the phosphorylation of H2AX has previously been reported but not in response to radiation. Indeed, it was demonstrated that commitment of the GABA receptor reduced stem cell proliferation via phosphorylation of H2AX and that ATM and DNA-PK catalyze this reaction [45]. However, looking carefully at this result, it could be observed that the inhibition of H2AX phosphorylation by an ATM-DNA-PK-specific inhibitor was not completely efficient, suggesting that other kinases were involved in the reaction, which is in agreement with the data presented here. Our experiments demonstrated that FGFR4 phosphorylates H2AX independently of ATM and that FGFR4 triggers a signaling cascade that eventually activates JNK1. Immunoprecipitation of H2AX along with JNK1 clearly indicates that H2AX is a substrate of this MAP kinase. This result is in agreement with the report that JNK1 phosphorylates H2AX in response to UV radiation [46]. This phosphorylation resulted in apoptosis.

In the early 1990s, the Fuks group reported the effect of FGF in the repair of radiation damage [1, 2]. Since then, numerous studies have demonstrated the radioprotective effect of several fibroblast growth factors, which may even have potential for clinical use as radioprotectors [3–7, 47, 48]. However, the underlying molecular mechanisms have never been completely characterized. Our results clearly link FGFR-dependent signaling to H2AX phosphorylation in response to radiation. Together, these results suggest that the presence of FGF will increase the phosphorylation of H2AX, leading in turn to more efficient DNA repair. The presence of FGF may trigger an adaptive response to irradiation, which in turn can explain the protective effect of FGF [17–21, 42, 44].

In contrast, deregulation of FGF signaling was shown to be associated with carcinogenesis. Mutations in *FGFR4* have been reported in lung cancer and breast cancer [49], and FGFR4 polymorphisms have been observed in squamous cell carcinoma [50] and breast cancer [51]. Finally, the FGFR4 Gly388Arg polymorphism has been shown to be a potential marker for prostate cancer development and progression [52, 53]. Interestingly, it seems that FGFR4 triggers the formation of γH2AX even in the absence of irradiation (Fig. 5b), which is in agreement with the oncogene-induced DNA damage model [54].

Although the mechanisms responsible for the different responses of cells to mutant FGFRs remain unclear, it has been postulated to be related to their ability to engage signaling pathways that can both promote and suppress proliferation, as well as by triggering negative feedback mechanisms [55]. As a consequence, several FGF signaling inhibitors were recently developed as new cancer therapeutic agents [56]. However, such inhibitors may trigger adverse reactions to radiation therapy and would have to be used with caution. Although H2AX and the PI3K-related kinases that phosphorylate H2AX have both been proposed as potential therapeutic targets [57–60], our results suggest the value of a multi-therapeutic approach that targets several kinases rather than only one.

### Electronic supplementary material

Below is the link to the electronic supplementary material.

Supplementary material 1 (PDF 1421 kb)
