# Supplementary material for: A kinome-targeted RNAi-based screen links FGF signaling to H2AX phosphorylation in response to radiation
Source: Cell Mol Life Sci. 2015 Apr 18;72(18):3559–73. doi: 10.1007/s00018-015-1901-7 (PMC4548013; doi:10.1007/s00018-015-1901-7)
Supplement: Supplementary file 1 — Supplementary material 1 (PDF 1421 kb) [file 18_2015_1901_MOESM1_ESM.pdf]

Legends for supplementary material

Figure S1. Validation of γH2AX readout in human keratinocytes. Keratinocyte cell line, HaCaT, was either irradiated at 2Gy or received a mock treatment. (A) Thirty minutes after treatment cells were fixed, washed and γH2AX foci formation was monitored by Cy3-labeled immunofluorescence and nuclei were stained with DAPI. Images were acquired with a Pathfinder OSA instrument developed by IMSTAR (Paris, France), an automated optical scanning system equipped with a high resolution (1300 x 1000 pixels) CCD camera. (B) Because of the nucleus-to-nucleus variability of γH2AX-specific fluorescence, we compare histograms of γH2AX-specific fluorescence at the single nucleus level from the entire cell population in triplicates rather than the averaged γH2AX signal.

Figure S2. Schematic representation of the BCR signaling. Kinases that were selected in the screening process are in red.

Figure S3. Schematic representation of the RAC signaling. Kinases that were selected in the screening process are in red.

Figure S4. The HaCaT cells were irradiated at 2 Gy with or without ATM specific inhibitor, then co-stained with DAPI and anti- γH2AX fluorescent antibodies. The γH2AX specific signal was quantified at single cell level.

Figure S1

A

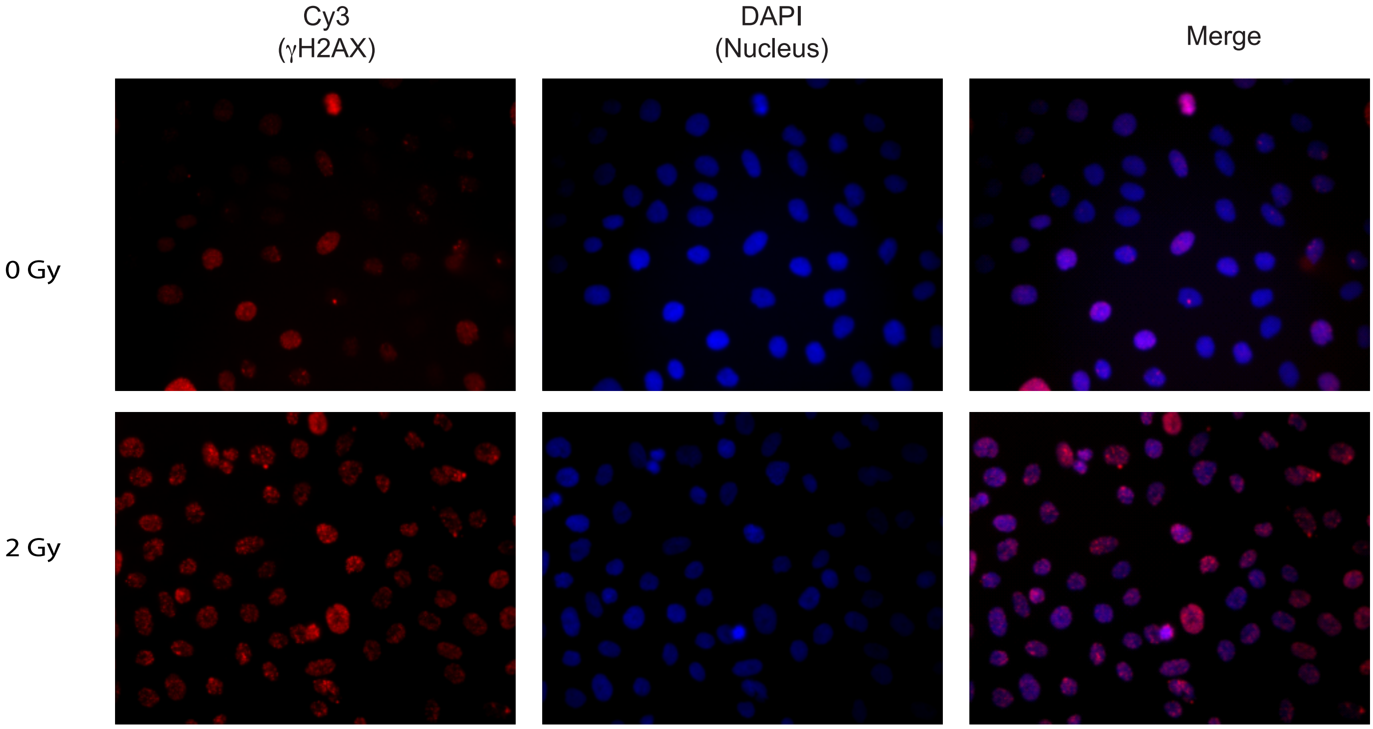
Cy3
(γH2AX)
DAPI
(Nucleus)
Merge
0 Gy
2 Gy

B

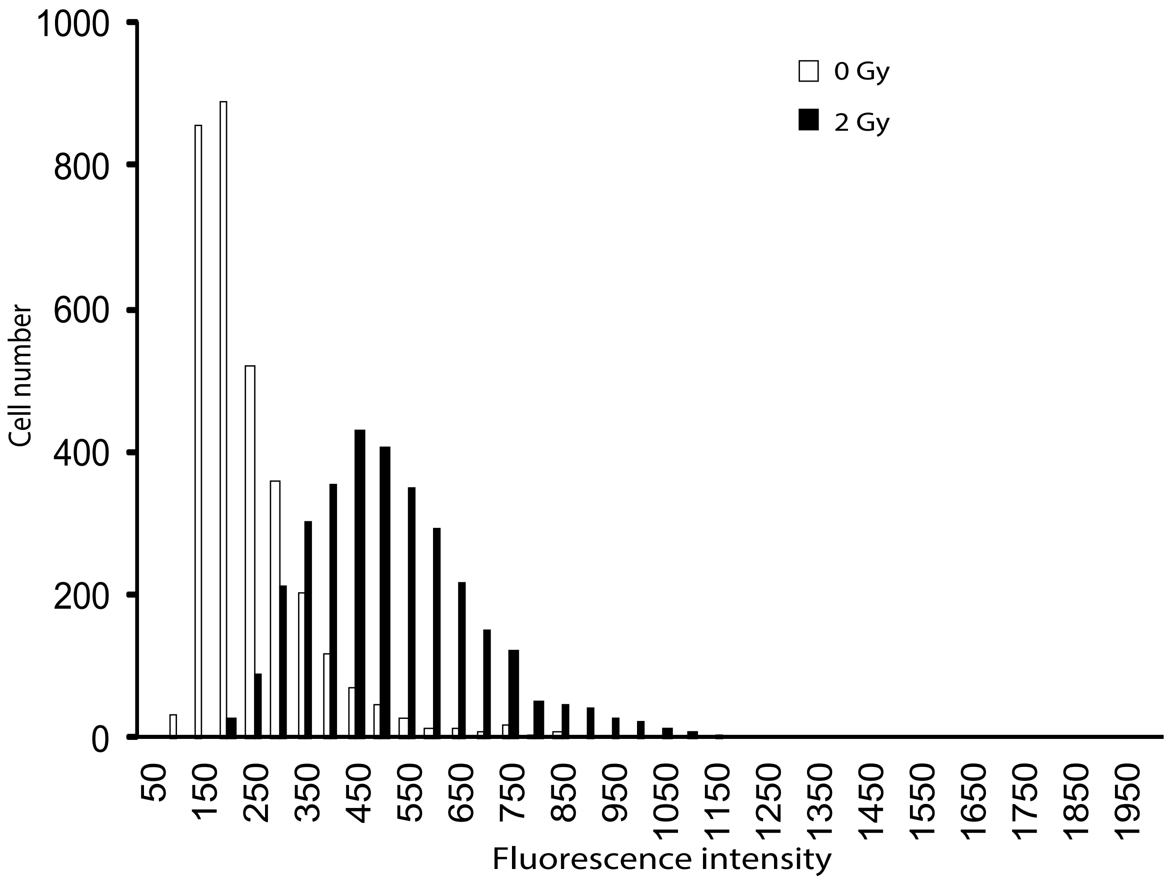
1000
800
600
400
200
0
Cell number
0 Gy
2 Gy
50
150
250
350
450
550
650
750
850
950
1050
1150
1250
1350
1450
1550
1650
1750
1850
1950
Fluorescence intensity

Figure S2

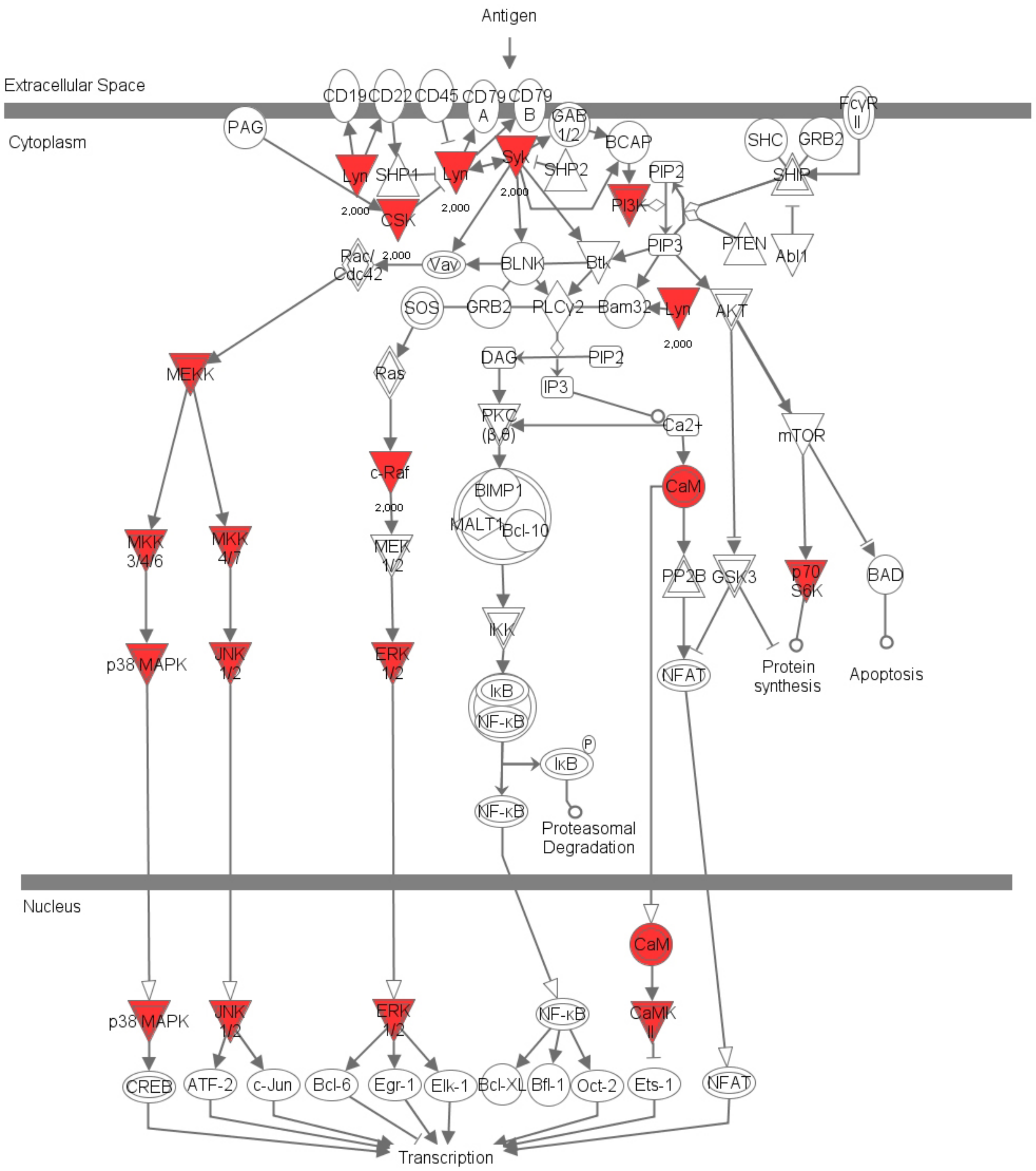
Antigen
Extracellular Space
Cytoplasm
Nucleus
CD19
CD22
CD45
CD79 A
CD79 B
PAG
GAB 1/2
BCAP
FcγR II
SHC
GRB2
SHIP
Lyn
SHP1
Lyn
Syk
SHP2
PI3K
PIP2
CSK
PTEN
Abl1
2,000
Rac/ Cdc42
Vav
BLNK
Btk
PIP3
SOS
GRB2
PLCγ2
Bam32
Lyn
AKT
MEKK
Ras
DAG
PIP2
IP3
PKC (β,θ)
Ca2+
mTOR
c-Raf
BIMP1
MALT1
Bcl-10
CaM
MKK 3/4/6
MKK 4/7
MEK 1/2
PP2B
GSK3
p70 S6K
BAD
IKK
p38 MAPK
JNK 1/2
ERK 1/2
NFAT
Protein synthesis
Apoptosis
IκB
NF-κB
P
Proteasomal Degradation
CaM
CaMK II
CREB
ATF-2
c-Jun
Bcl-6
Egr-1
Elk-1
Bcl-XL
Bfl-1
Oct-2
Ets-1
NFAT
Transcription

Figure S3

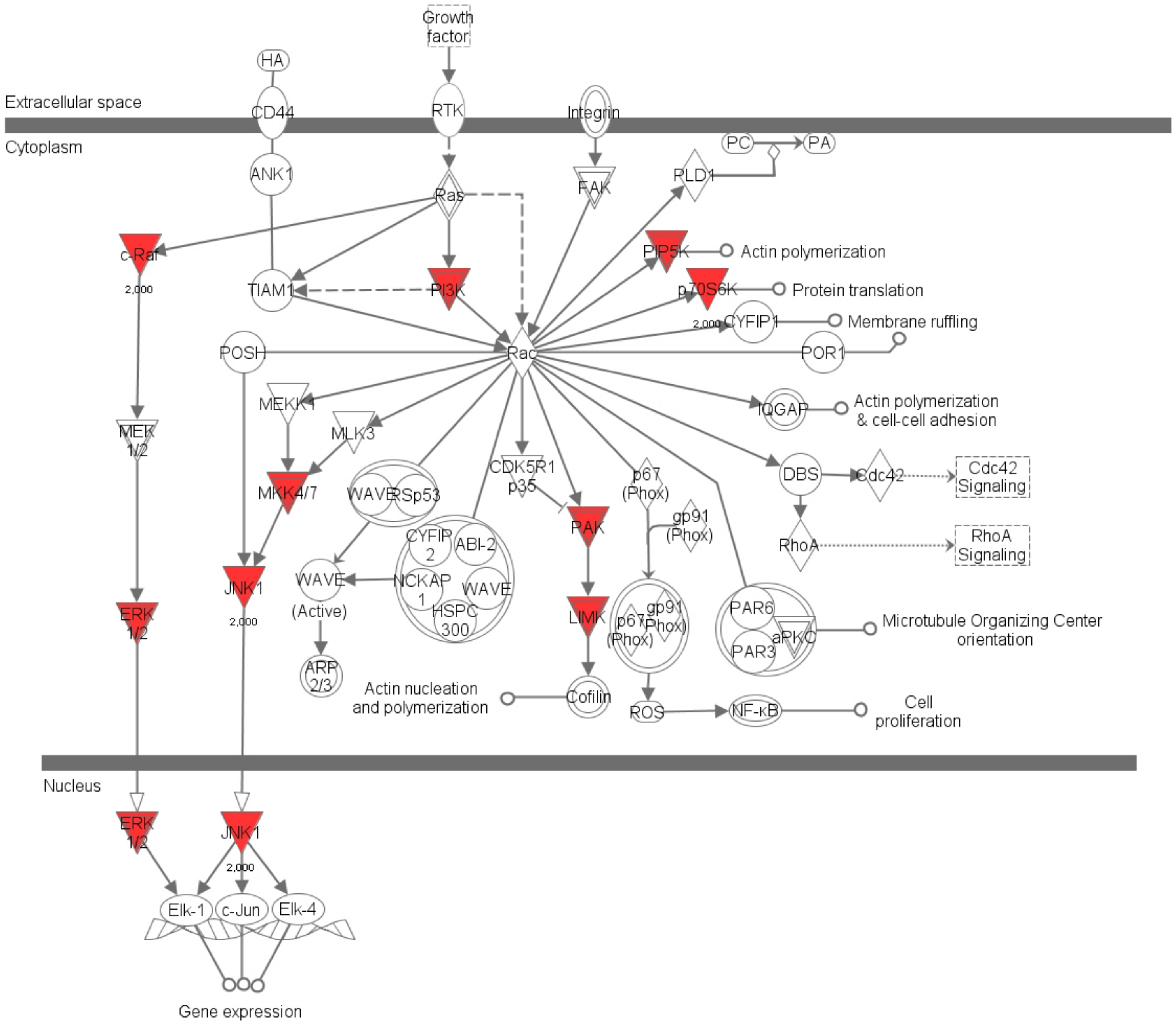
Growth factor
HA
CD44
RTK
Integrin
Extracellular space
Cytoplasm
PC
PA
ANK1
Ras
FAK
PLD1
c-Raf
2,000
PIP5K
Actin polymerization
TIAM1
PI3K
p70S6K
Protein translation
2,000
CYFIP1
Membrane ruffling
POSH
Rac
POR1
MEKK1
IQGAP
Actin polymerization & cell-cell adhesion
MEK 1/2
MLK3
CDK5R1 p35
p67 (Phox)
DBS
Cdc42
Cdc42 Signaling
MKK4/7
WAVE
RSp53
gp91 (Phox)
PAK
RhoA
RhoA Signaling
CYFIP 2
ABI-2
JNK1
2,000
WAVE (Active)
NCKAP 1
WAVE
HSPC 300
ERK 1/2
LIMK
p67 (Phox)
gp91 (Phox)
PAR6
PAR3
aPKC
Microtubule Organizing Center orientation
ARP 2/3
Actin nucleation and polymerization
Cofilin
ROS
NF-κB
Cell proliferation
Nucleus
ERK 1/2
JNK1
2,000
Elk-1
c-Jun
Elk-4
Gene expression

Figure S4

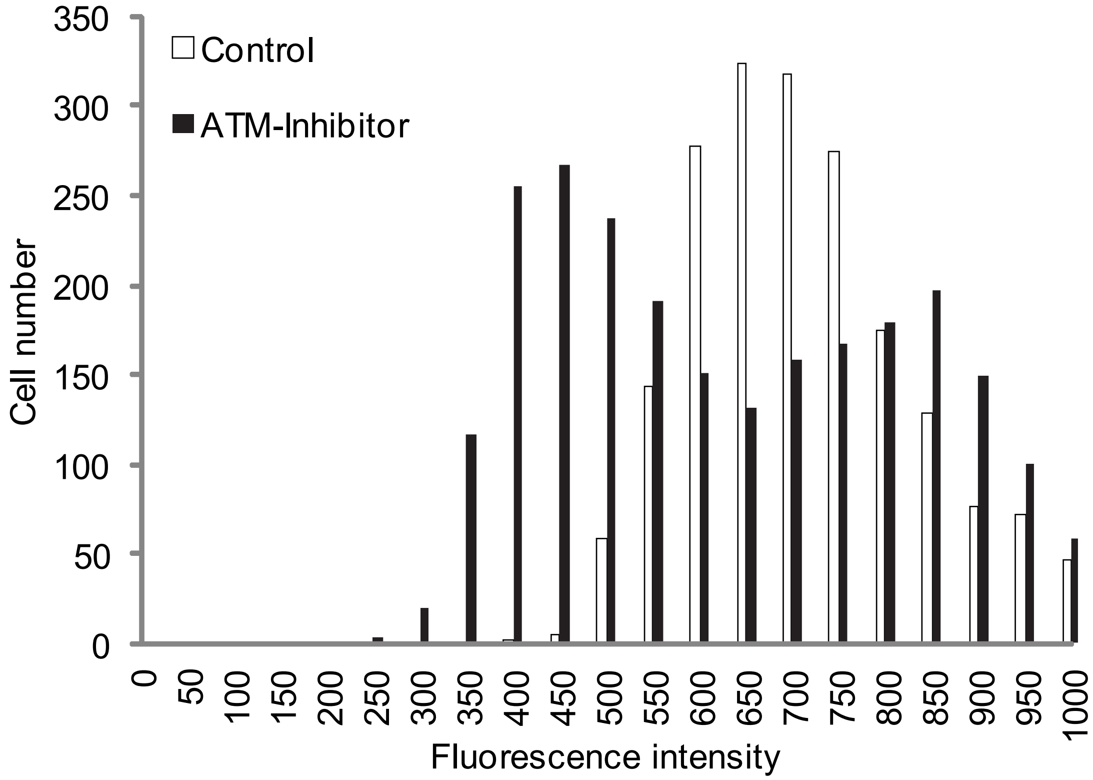

Control
ATM-Inhibitor
Cell number
350
300
250
200
150
100
50
0
0 50 100 150 200 250 300 350 400 450 500 550 600 650 700 750 800 850 900 950 1000
Fluorescence intensity
